# Dynamics of Conflicts in Wikipedia

**DOI:** 10.1371/journal.pone.0038869

**Published:** 2012-06-20

**Authors:** Taha Yasseri, Robert Sumi, András Rung, András Kornai, János Kertész

**Affiliations:** 1 Department of Theoretical Physics, Budapest University of Technology and Economics, Budapest, Hungary; 2 Computer and Automation Research Institute, Hungarian Academy of Sciences, Budapest, Hungary; Hungarian Academy of Sciences, Hungary

## Abstract

In this work we study the dynamical features of editorial wars in Wikipedia (WP). Based on our previously established algorithm, we build up samples of controversial and peaceful articles and analyze the temporal characteristics of the activity in these samples. On short time scales, we show that there is a clear correspondence between conflict and burstiness of activity patterns, and that memory effects play an important role in controversies. On long time scales, we identify three distinct developmental patterns for the overall behavior of the articles. We are able to distinguish cases eventually leading to consensus from those cases where a compromise is far from achievable. Finally, we analyze discussion networks and conclude that edit wars are mainly fought by few editors only.

## Introduction

New media such as the internet and the web enable entirely new ways of collaboration, opening unprecedented opportunities for handling tasks of extraordinary size and complexity. Such collaborative schemes have already been used to solve challenges in software engineering [1] and mathematics [2]. Understanding the laws of internet-based collaborative value production is of great importance.

Perhaps the most prominent example of such value production is Wikipedia (WP), a free, collaborative, multilingual internet encyclopedia [3]. WP evolves without the supervision of a pre-selected expert team, its voluntary editors define the rules and maintain the quality. WP has grown beyond other encyclopedias both in size and in use, having unquestionably become the number one reference in practice. Although criticism has been continuously expressed concerning its reliability and accuracy, partly because the editorial policy is in favor of consensus over credentials [4], independent studies have shown that, as early as in 2005, science articles in WP and Encyclopedia Britannica were of comparable quality [5]. As every edit and discussion post is saved and available, WP is particularly well suited to study internet-based collaborative processes. Indeed, WP has been studied extensively from different aspects including the growth of content and community [6], [7], coverage [8], [9] and evolution of the hyperlink networks [10]–[14], the extraction of semantic networks [15]–[17], linguistic studies [18]–[20], user reputation [21] and collaboration quality [22], [23], vandalism detection [24]–[26], and the social aspects of the editor community [27]–[32].

**Figure 1:**
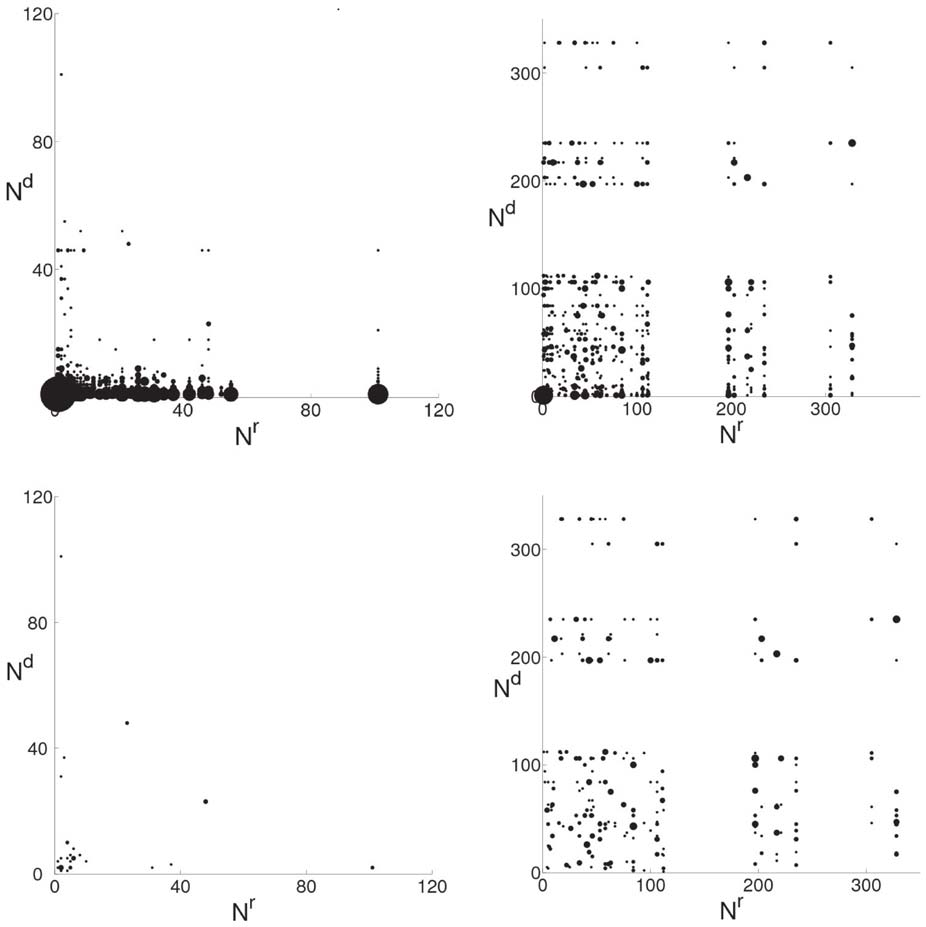
Revert and mutual revert maps of Benjamin Franklin (left) and Israel and the apartheid analogy (right). Diagrams in upper row show the map of all reverts, whereas only mutual reverts are depicted on the diagrams in the lower row. 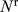 and 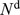 are the number of edits made by the reverting and reverted editors respectively. Size of the dots is proportional to the number of reverts by the same reverting and reverted pair of editors.

Usually, different editors constructively extend each other’s text, correct minor errors and mistakes until a consensual article emerges – this is the most natural, and by far the most common, way for a WP entry to be developed [33]. Good examples include (WP articles will be cited in typewriter font throughout the text) Benjamin Franklin, Pumpkin or Helium. As we shall see, in the English WP close to 99% of the articles result from this rather smooth, constructive process. However, the development of WP articles is not always peaceful and collaborative, there are sometimes heavy fights called *edit wars* between groups representing opposing opinions. Schneider et al. [34] estimated that in the English WP, among the highly edited or highly viewed articles (these notions are strongly correlated, see [35]), about 12% of discussions are devoted to reverts and vandalism, suggesting that the WP development process for articles of major interest is highly contentious. The WP community has created a full system of measures to resolve conflict situations, including the so called “three revert rule” (see Wikipedia:Edit warring), locking articles for non-registered editors, tagging controversial articles, and temporal or final banning of malevolent editors. It is against this rich backdrop of explicit rules, explicit or implicit regulations, and unwritten conventions that the present paper undertakes to investigate a fundamental part of the collaborative value production, how conflicts emerge and get resolved.

**Figure 2:**
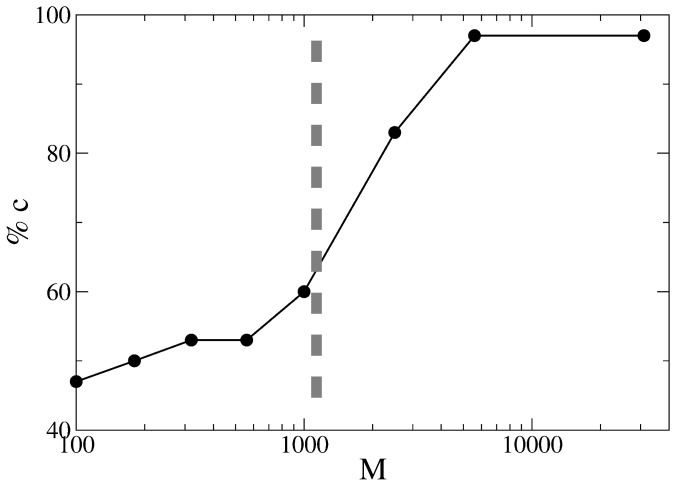
The percentage of true positives in detection of controversial articles compared to human judgment at different values of *M* 
 **.** A threshold of 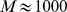 for controversiality is selected according to this diagram.

The first order of business is to construct an automated procedure to identify controversial articles. For a human reader the simplest way to do so is to go to the discussion (talk) pages of the articles, which often show the typical signatures of conflicts as known from social psychology [36]. The length of the discussion page could already be considered a good indicator of conflict: the more severe the conflict, the longer the talk page is expected to be (this will be shown in detail later). However, this feature is very language dependent: while conflicts are indeed fought out in detail on discussion pages in the English WP, German editors do not use this vehicle for the same purpose. Moreover, there are WPs, e.g. the Hungarian one, where discussion pages are always rather sparse, rarely mentioning the actual arguments. Clearly the discussion page alone is not an appropriate source to identify conflicts if we aim at a general, multi-lingual, culture-independent indicator.

Conflicts in WP were studied previously both on the article and on the user level. Kittur et al. [37], [38] and Vuong et al. [39] measured controversiality by counting the “controversial” tag in the history of an article, and compared other possible metrics to that. It should be noted, however, that this is at best a one-sided measure as highly disputed pages such as Gdansk or Euthanasia in the English WP lack such tags, and the situation is even worse in other WPs. In [38], different page metrics like the number of reverts, the number of revisions etc. were compared to the tag counts and in [39] the number of deleted words between users were counted and a “Mutual Reinforcement Principle” [40] was used to measure how controversial a given article is. Clearly, there are several features of an article which correlate with its controversiality, making it highly non-trivial to choose an appropriate indicator. Some papers try to detect the negative “conflict” links between WP editors in a given article and, based on this, attempt to classify editors into groups. The main idea of the method used by Kittur et al. [38] is to relate the severity of the conflict between two editors to the number of reverts they carry out on each other’s versions. In a more recent study [41], [42], Brandes et al. counted the number of deleted words between editors and used this as a measure of controversy.

**Figure 3:**
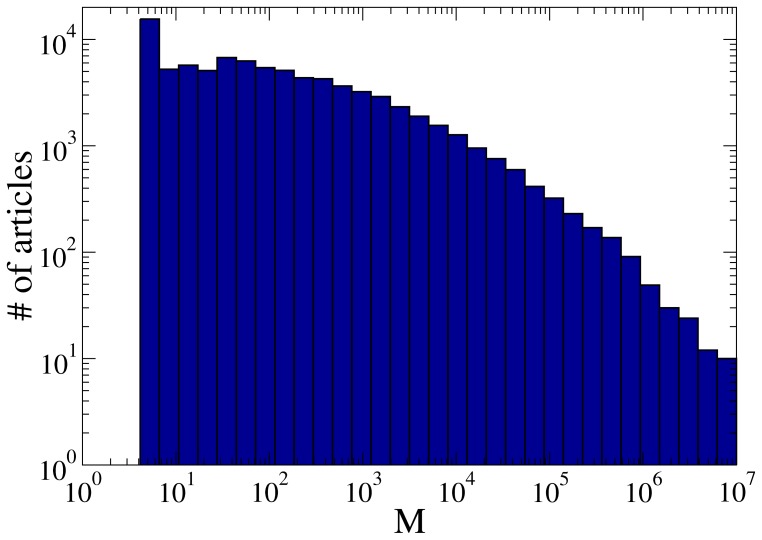
Histogram of articles according to their controversiality measure 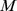
 **.** There are some 84 k articles with 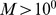, 12 k controversial articles with *M* 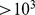, and less than 100 super-controversial articles with 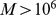.

There is no question that reverting a part of an article expresses strong disagreement, but sometimes this is just related to eliminating vandalized texts, while in other cases it is related to conflict about the contents of the article. Here we are interested in the second case and it will be one of our goals to distinguish between deeper conflict and mere vandalism. Beyond identifying conflict pages and edit wars, we aim at relating different properties of the articles to their level of controversiality. In the Methods section we describe the dataset, summarize our conflict identification method, and relate it to other measures proposed in the literature. In the main body of the paper we analyze the temporal evolution of conflicts both on the micro and the macro timescales and, based on that, we try to categorize them.

**Figure 4:**
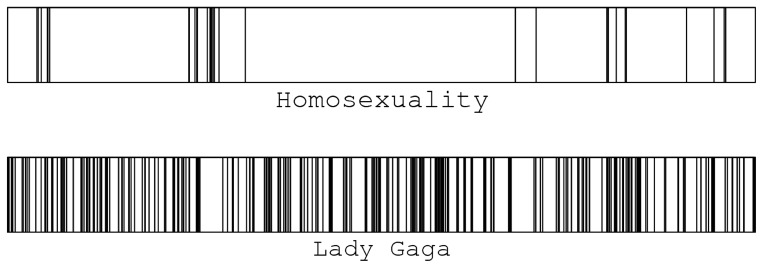
Temporal edit patterns of Lady Gaga and Homosexuality during a one month period (12/2009). The horizontal axis is time, each vertical line represents a single edit. Despite the large differences in average time intervals between successive edits, the bursty editing pattern is common to both cases.

## Methods

To analyze edit wars in WP first we need to be able to detect the articles where significant debates occur. For the human viewer of page histories it is evident that an article such as Liancourt Rocks, discussing a group of small islets claimed by both Korea and Japan, or the article on Homosexuality were the subject of major edit wars. Yet articles with a similar number or relative proportion of edits such as Benjamin Franklin or Pumpkin were, equally evidently to the human reader, developed peacefully. For our conflict detection method (previously reported in [43], [44]), similar to most pattern recognition tasks such as speech or character recognition, we take human judgment to be the gold standard or “truth” against which machine performance is to be judged. How human judgment is solicited is discussed in Text S1.

**Figure 5:**
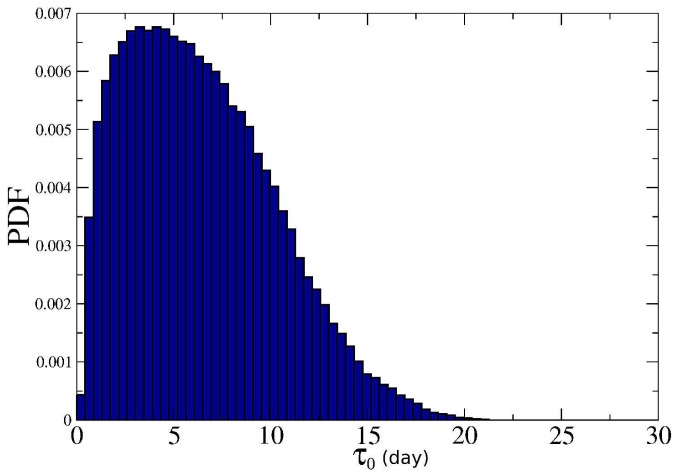
PDF of the average time 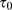 between two successive edits of articles measured in days. In any two week period most of the articles are edited twice or more.

**Figure 6:**
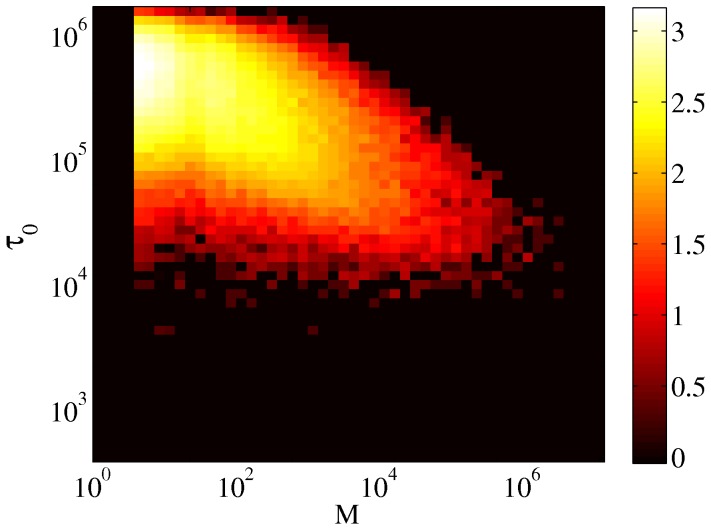
Scatter plot of the average time interval between successive edits and the controversy measure. Color coding is according to logarithm of the density of points. The correlation coefficient 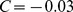.

The whole structured dataset and the implementation of the ranking algorithm described below, along with the raw results, are available at *WikiWarMonitor* webpage: http://wwm.phy.bme.hu.

**Figure 7:**
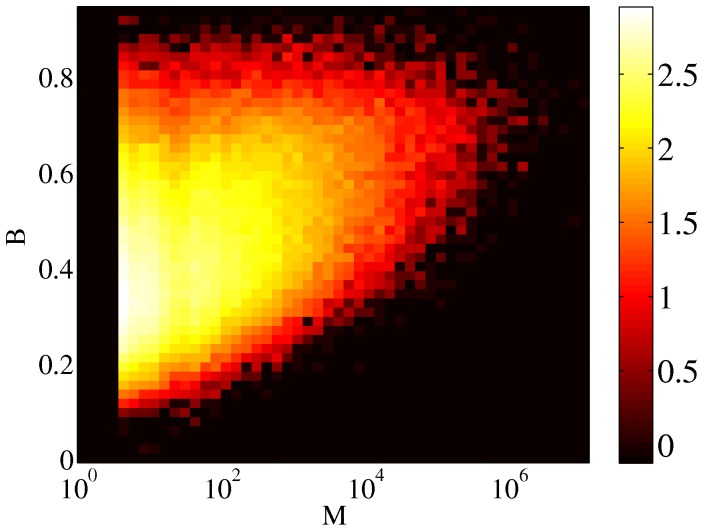
Scatter plot of burstiness and the controversy measure. Color coding according to logarithm of the density of points. The correlation coefficient 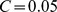.

### Dataset

Our analysis is based on the January 2010 dump of the English WP [45], which contains all the versions of all pages up to that date. The dataset originally contains 3.2 M articles, but we have filtered out all short (less than 1,000 characters) and evidently conflict-free (less than 100 edits) articles, leaving a final set of around 223 k articles.

### Detecting Edit Wars

Our detection method is entirely based on statistical features of edits and is therefore independent of language characteristics. This makes possible both inter-cultural comparisons and cross-language checks and validation.

**Figure 8:**
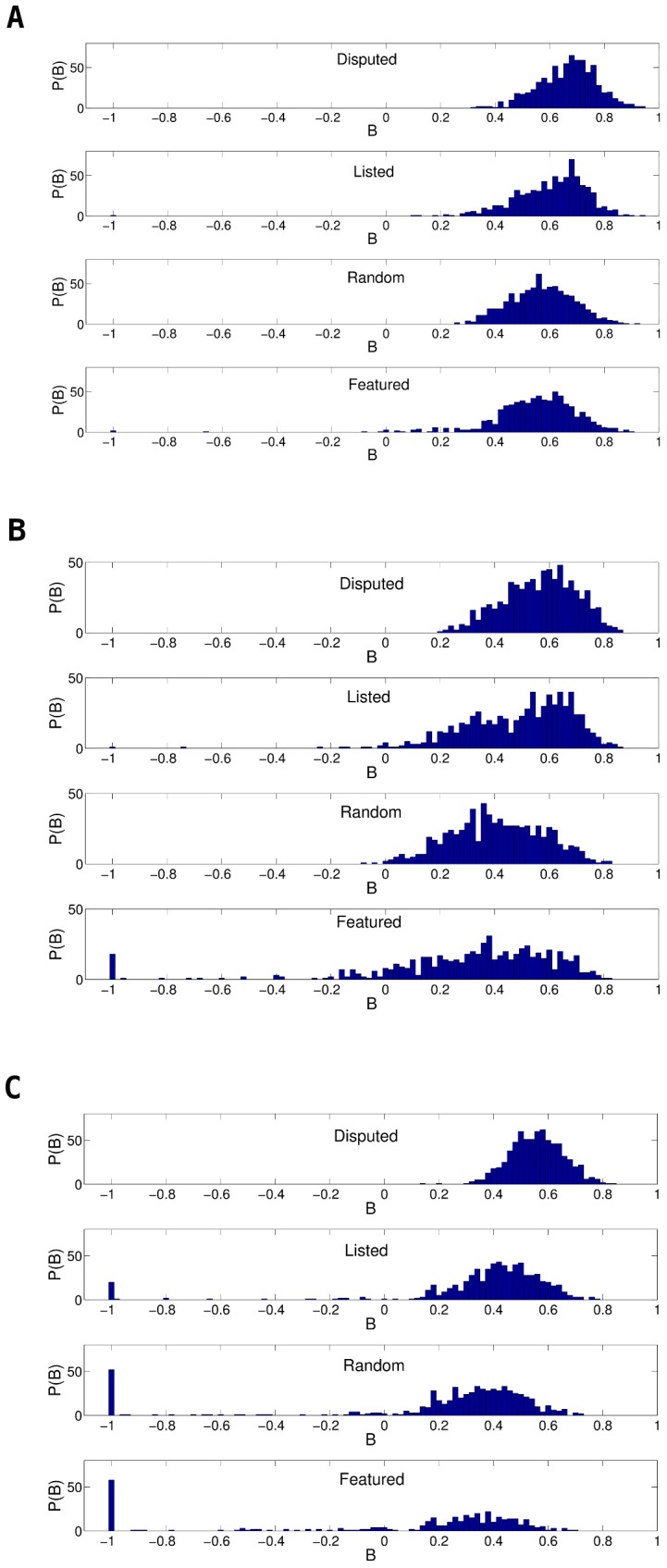
Histogram of burstiness of A) all edits, B) reverts, and C) mutual reverts for four classes of articles. High controversy (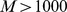, topmost panels),listed as controversial (2nd panels), randomly selected (3rd panels), and featured articles (bottom panels).

#### Revert maps

To detect reverts we calculated the MD5 [46] hash for each revision, and reverts were identified by comparing the hash of different revisions. Let 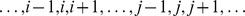 be stages in the history of an article. If the text of revision 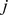 coincides with the text of revision 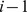, we considered this a revert between the editor of revision 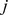 and 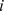 respectively. Let us denote by 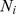 the total number of edits in the given article of that user who edited the revision 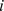. We characterize reverts by pairs 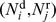, where 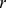 denotes the editor who makes the revert, and 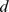 refers to the reverted editor (self-reverts are excluded). Figure 1 represents the revert map of the non-controversial Benjamin Franklin and the highly controversial Israel and the apartheid analogy articles. Each mark corresponds to one or more reverts. The revert maps already distinguish disputed and non-disputed articles, and we can improve the results by considering only those cases where two editors revert each other mutually, hereafter called mutual reverts. This causes little change in disputed articles (compare the right panels of Figure 1 but has great impact on non-disputed articles (compare left panels).

**Figure 9:**
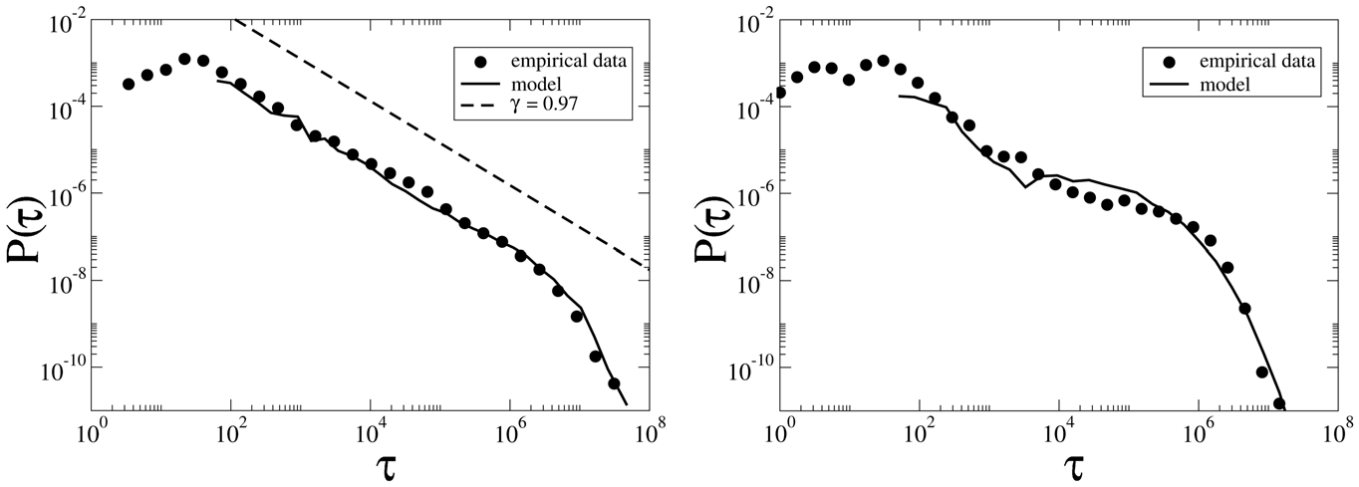
PDF of intervals between two successive edits on an article (in seconds) for two samples of highly/weakly disputed articles (left/right panel). Each sample contains 20 articles and the average 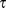 for all articles is about 10 hours. Circles are empirical data and solid lines are model fit, with values 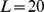, 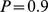 and 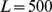, 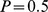 respectively for disputed and non-disputed samples. The dashed line in the left panel is the power law with exponent 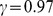.

#### Controversy measure

Based on the rank (total edit number within an article) of editors, two main revert types can be distinguished: when one or both of the editors have few edits to their credit (these are typically reverts of vandalism since vandals do not get a chance to achieve a large edit number, as they get banned by experienced users) and when both editors are experienced (created many edits). In order to express this distinction numerically, we use the *lesser* of the coordinates 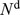 and 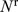, so that the total count includes vandalism-related reverts as well, but with a much smaller weight. Thus we define our raw measure of controversiality as.

**Figure 10:**
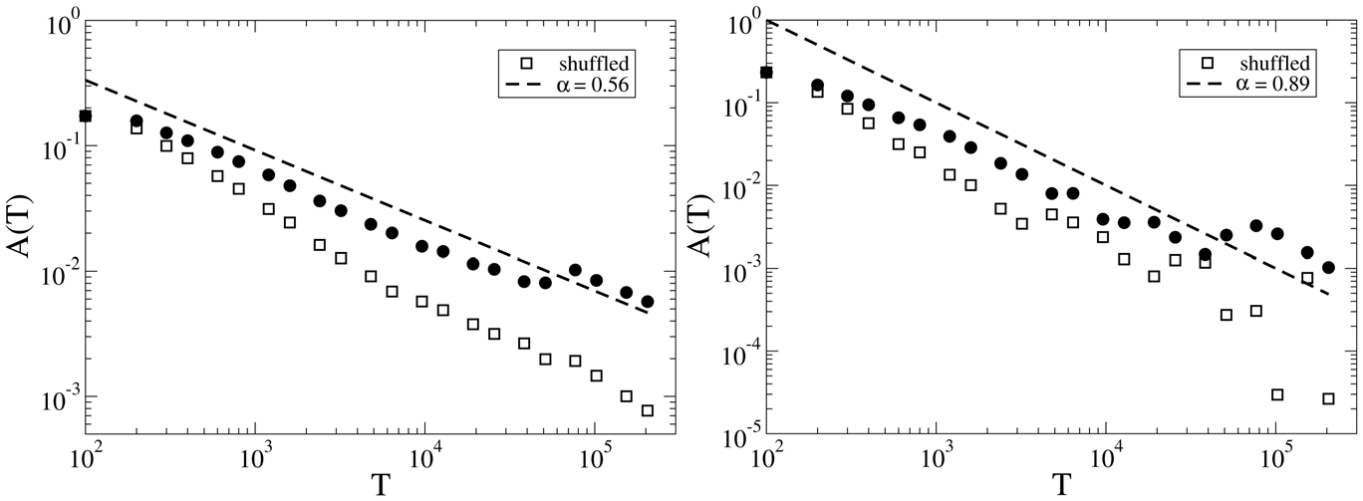
Autocorrelation function of edits sequences for two samples of highly/weakly disputed articles (left/right panel). Circles are for the original sequences, empty squares correspond to the shuffled sequences. Dashed lines are power-law fits.



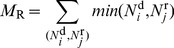
(1)Once we developed our first auto-detection algorithm based on 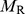, we iteratively refined the controversial and the noncontroversial seeds on multiple languages by manually checking pages scoring very high or very low. In this process, we improved 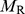 in two ways: first, by multiplying with the number of editors 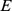 who ever reverted mutually (the larger the armies, the larger the war) and define 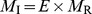 and second, by censuring the topmost mutually reverting editors (eliminating cases with conflicts between two persons only). Our final measure of controversiality 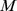 is thus defined by
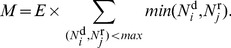
(2)


#### Evaluation and accuracy

One conceptually easy (but in practice very labor-intensive) way to validate 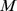 is by simply taking samples at different 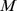 values and counting how many controversial pages are found (see Figure 2), considering human judgment as the “truth”. We have checked this measure for six different languages and concluded that its overall performance is superior to other measures [44].

**Figure 11:**
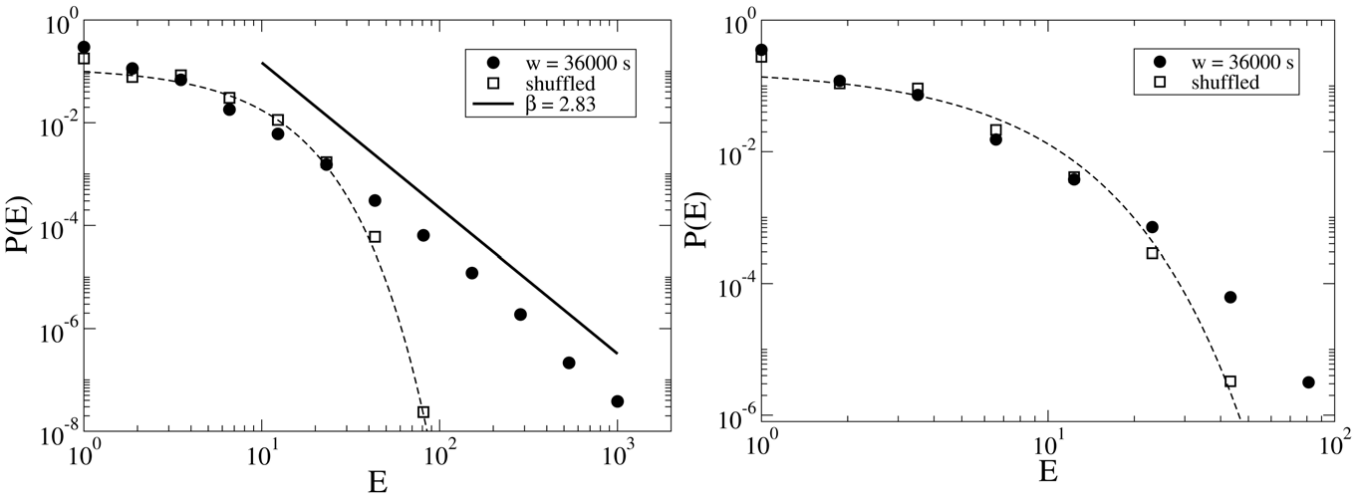
Distribution of 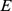 for two samples of highly/weakly disputed articles (left/right panel). Circles are for the original sequences, whereas empty squares correspond to the shuffled sequences. Dashed lines are exponential fits to the 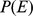 for shuffled data and solid line in the left panel is a power-law with 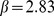.

## Results and Discussion

Having validated the 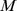-based selection process, we can start analyzing the controversial and peaceful articles from a variety of perspectives. We calculated 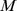 for all the articles in the sample – a histogram is shown in Figure 3. The primary observation here is that the overall population of controversial articles is very small compared to the large number of total articles. Out of our sample of 233 k articles, there are some 84 k articles with nonzero 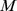, and only about 12 k with 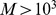. The number of super-controversial articles with 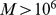 is less than 100.

**Table 1:** Scaling exponents for the two samples of controversial and peaceful articles, and users.

	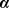	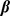	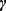
Low M articles	0.89±0.02	–	–
High M articles	0.56±0.01	2.83±0.06	0.97±0.01
Users	0.46±0.01	3.05±0.03	1.44±0.01

Edit patterns of controversial articles and activity patterns of users show all the expected features of bursty correlated processes.

We mention in passing that the topical distribution of the controversial issues differs significantly spatially (across different language editions of WP): for example, soccer-related issues are massively controversial in the Spanish WP but not elsewhere. There are flashpoints common to all languages and cultures, in particular religious and political topics, but we leave the detailed cross-cultural analysis for another occasion [47]. Here we focus on the temporal aspects of conflict (based, unless specifically mentioned otherwise, on the English WP), first at the *micro-dynamic* level (hours, days, and weeks), and next on a macro timescale (the lifetime of the article, typically measured in years) to see the *overall patterns* of conflicts.

**Figure 12:**
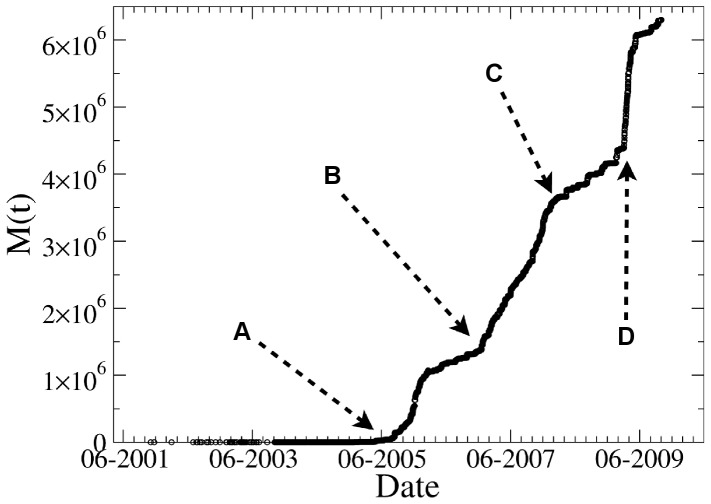
Time evolution of the controversy measure of Michael Jackson. A: Jackson is acquitted on all counts after five month trial. B: Jackson makes his first public appearance since the trial to accept eight records from the Guinness World Records in London, including *Most Successful Entertainer of All Time.* C: Jackson issues *Thriller 25*. D: Jackson dies in Los Angeles.

### Micro-dynamics of Conflicts

Once we have a reliable measure of controversiality, not only can we find and rank controversial issues in WPs, but we actually begin to see important phenomena and common characteristics of wars and disputes. Here we report our findings on the temporal characteristics of edits on high and low controversiality pages. We make use of the fact that in the WP dump a timestamp with one second precision is assigned to each edit. One month of activity (the time-line of all edits irrespective of who performed them) on two sample articles are depicted in Figure 4.

West et. al. [48] and Adler et. al. [26] have developed vandalism detection methods based on temporal patterns of edits. In both studies the main assumption is that offensive edits are reverted much faster than normal edits, and therefore, by considering the time interval between an arbitrary edit and its subsequent reverts, one can classify vandalized versions with high precision.

**Figure 13:**
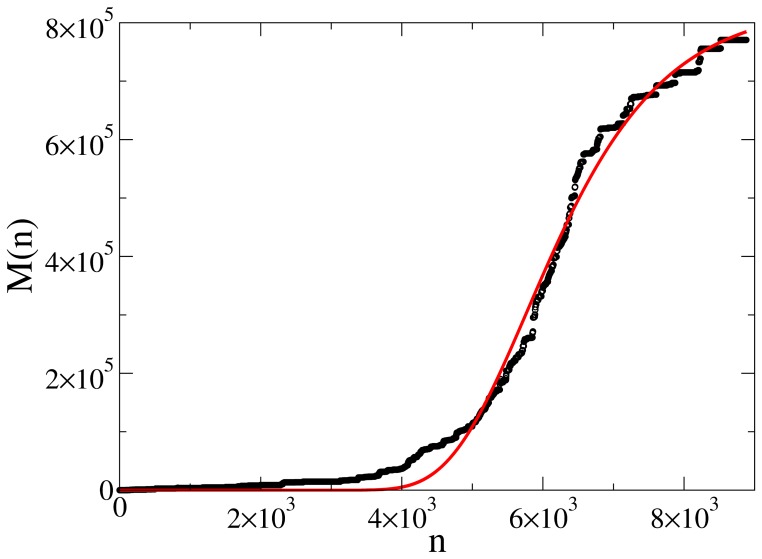
Evolution of controversy measure with number of edits of Jyllands-Posten Muhammad cartoons controversy, with Gompertz fit shown in red. The initial rapid growth in 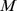 tends to saturate, corresponding to the reaching to consensus.

**Figure 14:**
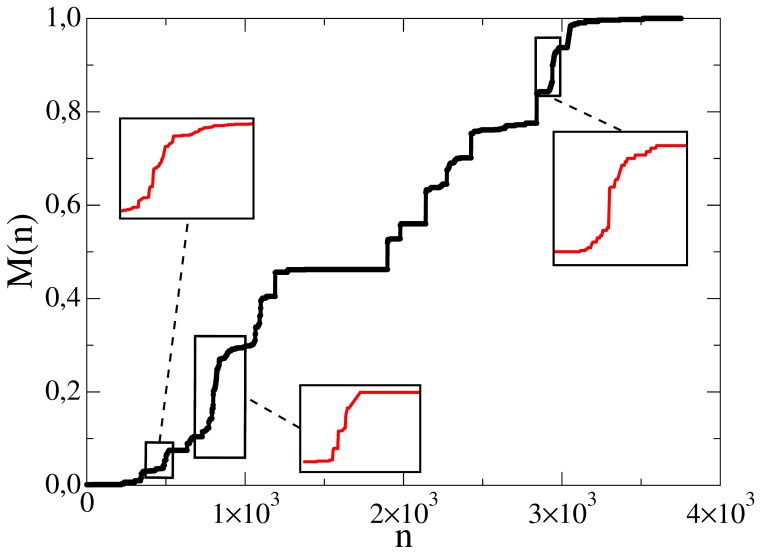
Evolution of controversy measure with number of edits of of Iran – the insets depict focuses of some of the local war periods. 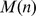
 is normalized to the final value 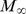. Cycles of peace and war appear consequently, activated by internal and external causes.

#### Edit frequency

Most of the articles are frequently edited. Figure 5 shows the empirical probability density function of the average time 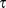 between two successive edits. As already noted in [35] edit frequency also depends on the controversiality of a page, and one expects higher edit frequency for more controversial pages. However, as Figure 6 makes clear, the correlation is quite weak (correlation coefficient 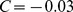).

**Figure 15:**
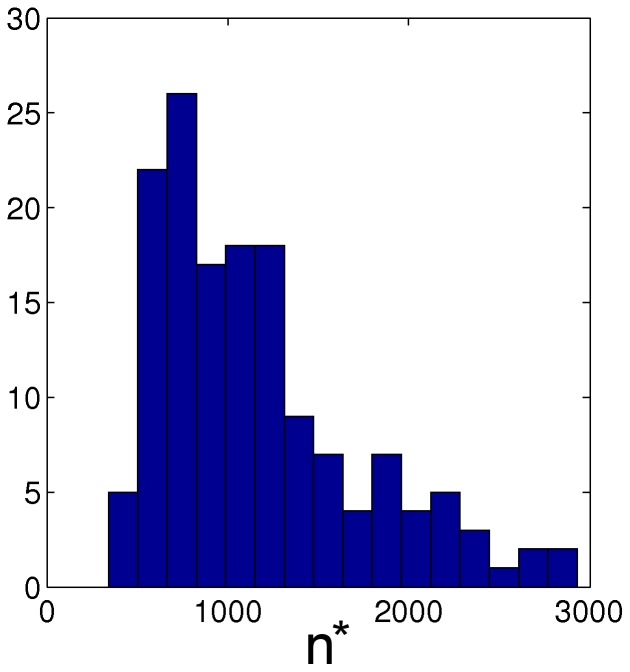
Length of peacful periods. Histogram of number of edits between two successive war periods for a selected sample of 44 articles which are not driven by external events. The average value of 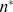 is 1300 edits.

#### Burstiness

It is clear that edits are clustered in a way that there are many edits done in a rather short period, followed by a rather long period of silence. This feature is known in the literature as *burstiness* [49], [50], and is quantified based on the coefficient of variation by a simple formula as
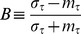
(3)where 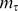 and 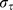 denote respectively the mean and standard deviation of the interval 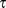 between successive edits. We have calculated 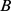 for all the articles in the sample, considering all the edits made on them by any user. As it can be seen from Figure 7, overall burstiness of edits correlates rather weakly with controversiality (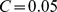).

**Figure 16:**
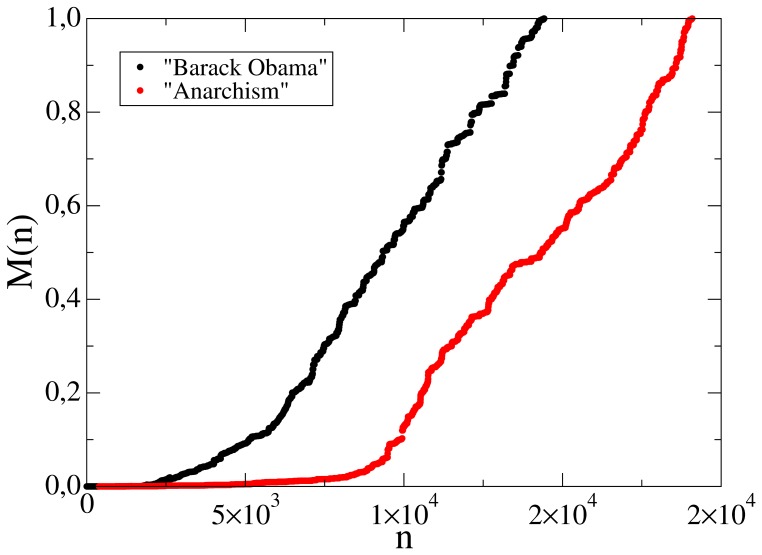
Evolution of controversy measure with number of edits of Anarchism and Barack Obama. 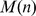
 is normalized to the final value 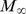. There is no consensus even for a short period and editorial wars continue nonstop.

To see the impact of controversiality on burstiness we calculated 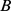 for different groups of articles separately: *Disputed* articles 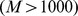, *Listed* articles coming from the List of controversial articles in WP [51], *Random*ly selected articles, and *Featured* articles (assumed to be least controversial given WPs stringent selection criteria for featuring an article). The histograms in Figure 8(A) show the PDF of 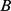 in these four classes. As can be seen, the peaks are shifted to the right (higher 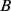) for more controversial articles, but not strongly enough to base the detection of controversy on burstiness of editorial activity alone. Reverting is a useful tool to restore vandalized articles, but it is also a popular weapon in heated debates. Figure 8(B) shows the distribution of 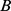 calculated not for all edits, but for reverts alone: the shift is now more marked. Finally, we considered an even stronger form of warfare: *mutual reverts*. It is evident that the temporal pattern of mutual reverts provides a better characterization of controversiality than that of all edits or all reverts, and the very visible shift observed in Figure 8(C) constitutes another, albeit less direct, justification of our decision to make mutual reverts the central element in our measure of controversiality.

**Figure 17:**
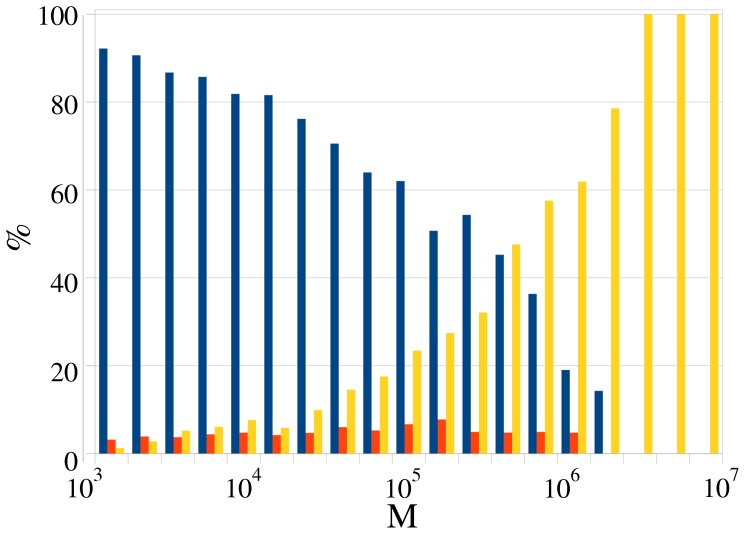
Relative share of each category at different *M* . Blue: category (a), consensus. Red: category (b), multi-consensus. Yellow: Category (c), never-ending war. For the precise definition of each category see the main text.

**Figure 18:**
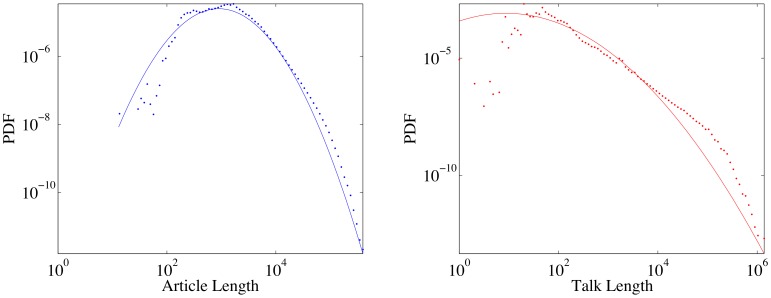
Length distribution of articles and talk pages with log-normals fits. The distribution of articles length is better described by a log-normal distribution compared to the talk length distribution, which tends to be more like a power-law.

To gain a better understanding of the microdynamics of edit wars, we selected two samples of 20 articles each, extracted from a pool of articles with average successive edit time intervals of 10 hours 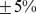. One sample contains the most controversial articles in the pool with 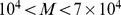, whereas the other one contains the most peaceful articles with 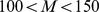. The probability distribution of time 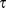 between edits for these samples is shown in Figure 9. Both samples have a rather fat-tailed distribution with a shoulder in the distribution (as observed both in the empirical data and the model calculation), indicating that a characteristic time, 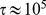 seconds (one day), is present in the system. However, only the sample consisting of controversial articles displays a clear power-law distribution, 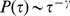,with 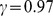. All exponents were calculated by applying the Gnuplot implementation [52] of the nonlinear least-squares Marquardt-Levenberg algorithm [53] on the log-binned data with an upper cut-off to avoid system size effects.

**Figure 19:**
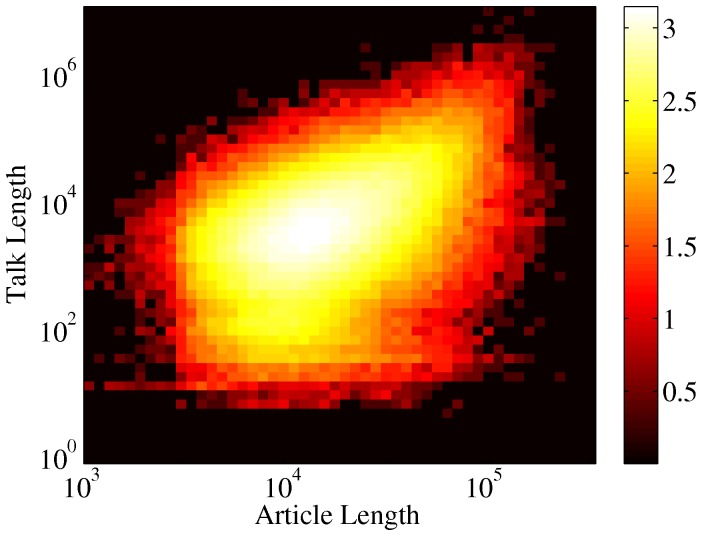
Scatter plot of talk page vs. article length. Color coding is according to logarithm of the density of points. The correlation between the length of the article and the corresponding talk page is weak, 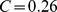.

**Figure 20:**
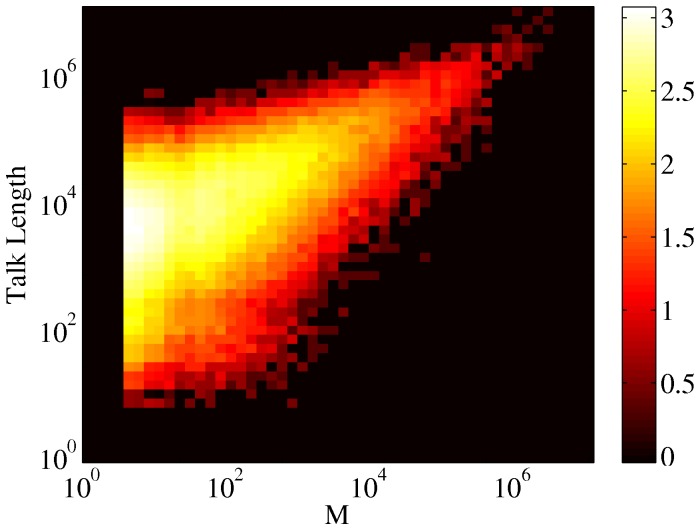
Scatter plot of talk page length vs. 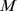
 **.** Color coding is according to logarithm of the density of points. There is a rather clear correlation, 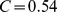 between the length of the talk page and the controversality of the article.

To fit the data depicted in Figure 9, we used a model based on a queuing mechanism introduced in [50] and further developed in [54]. Here we briefly explain its basis and how we use it to model our empirical findings. Let us assume that there is a list of 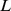 articles and there is only one editor (mean-field approximation) who edits at each step once. With probability 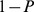, the editor selects the article to edit from the list randomly and with no preference among 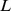 choices. With probability 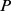 the articles will be selected according to a priority 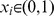 which is assigned randomly to the 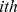 article after each edit on it. The key parameters are 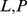 and the real time 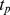 associated to the model time step. Controversial articles are fitted well by 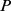 close to 1 and small 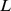. Uncontroversial articles fit with large 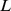 and smaller 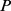, in nice agreement with the real situation, where editors tend to edit a few controversial articles more intentionally and many peaceful articles in a more or less uncorrelated manner with no bias and memory. To check the validity of the model, we calculated the ratio of the number of controversial articles (with 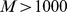) to the rest of the articles (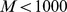) to be 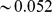, which is in nice agreement with the fitting model parameters, 20/500 = 0.04.

Another important characteristic quantity is the autocorrelation function 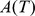. To calculate it, first we produce a binary series of 0/1 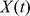 similar to the one in Figure 4. Then 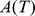 is computed simply as
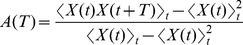
(4)


**Figure 21:**
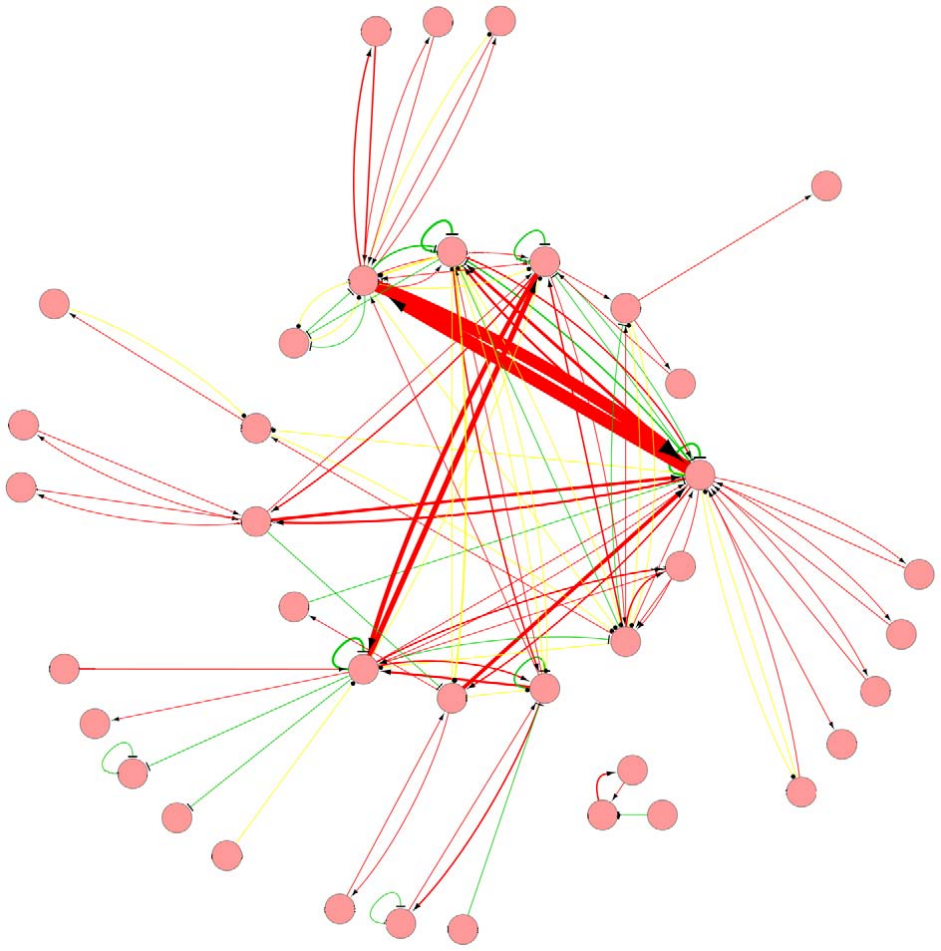
Network representation of editors’ interactions in the discussion page of Safavid dynasty. Each circle is an editor, red arrows represent comments opposing the target editor, T-end green lines represent positive comments (agreeing with the other editor), and yellow lines with round end represent neutral comments. Line thickness is proportional to the number of times that the same interaction occurs. Data based entirely on subjective assessments (manual review).

where 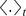 stands for the time average over the whole series. 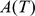 for the same samples of controversial and peaceful articles are shown in Figure 10. We calculate the same quantity for a shuffled sequence of events as a reference. The shuffled sequence has the same time interval distribution as the original sequence, but with a randomized order in the occurrence of events. In both cases, a power-law of 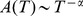 describes 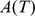 very well. Usually it is assumed that slow (power law) decay of the autocorrelation function is an indicator for long time memory processes. However, if independent random intervals taken from a power law distribution separate the events, the resulting autocorrelation will also show power law time dependence [55], [56]. Assuming that the exponent of the independent inter-event time distribution is 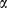 and the exponent of the decay of the correlation function is 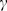, we have the relationship 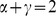. Deviations from this scaling law reflect intrinsic correlations in the events.

**Figure 22:**
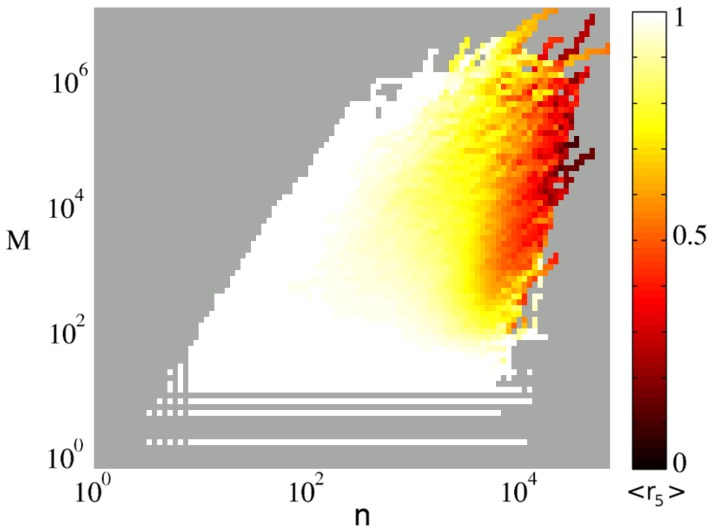
Average 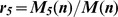, color coded for different *M* 
’s and *n* 
’s. For a wide range of articles and in a long time of their lives 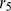, the relative contribution of the top 5 most reverting pair of editors, is very close to 1, making clear the important role of the top 5 pairs of fighting editors.

There is another measure which indicates long time correlations between the events even more sensitively. Take a period to be bursty if the time interval between each pair of successive edits is not larger than 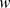, and define 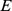 as the number of events in the bursty periods. If events in the time series are independent and there is no memory in the system (i.e. in a Poisson process), one can easily show that 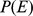 should have an exponential decay, whereas in the presence of long range memory, the decay is in the form of 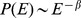 [56]. In Figure 11, 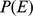 is shown for samples of highly controversial and peaceful articles. In the high controversy sample a well defined slope of -2.83 is observed, while in the low controversy sample edits are more independent and 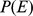 is very close to the one obtained for the shuffled sequence. Note that by shuffling the sequence of time intervals, all the correlations are eliminated and the resulting sequence should mimic the features of an uncorrelated occurrence of the intervals.

The same measurements are performed for a sample of users, see Figure S1 in Supporting Information. In Table.1, a summary of the scaling exponents for the both article samples and users is reported.

The simplest explanation of these results is to say that conflicts induce correlations in the editing history of articles. This can already be seen in Figure 10, where shuffling influences the decay of the autocorrelation functions much more for high-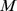 articles than for low-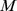 ones. For the more sensitive measure 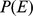 the original and the shuffled data are again quite close to each other for the low-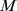 case, while a power-law type decay can be observed in the empirical data for high-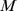 articles.

### Overall Patterns of Conflicts

Before we can consider the macro-scale evolution of 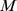 (during the entire life of the article), we need to make an important distinction between endo- and exogenous causes of conflict. Our principal interest is with endogenous forces, which originate in internal sources of conflict and disagreement, but it cannot be denied that in a significant number of cases conflicts are occasioned by some exogenous event, typically some recent development related to the real-world subject of the article rather than to its text (see Figure 12 for some examples).

#### Categorization

In the presence of significant exogenous events one can best follow the increase of 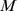 as a function of time 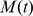, but if endogenous edits dominate (as is the case with most science articles and bibliographies of persons long dead) it is more natural to trace 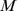 as a function of the number of edits on the article 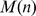 because temporal frequency of edits changes from time to time and from article to article, due to many different known and unknown causes [57], [58]. Since exogenous factors are completely unpredictable, in the following section we try to categorize articles according to 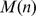.

Even if we restrict attention to endogenous growth, very different patterns can be observed in the evolution of 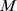, depending not just on the current controversiality of a subject (by definition, 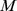 never decreases except for small truncation effects due to changes in who are the most engaged pair of reverters), but also on the micro-dynamics of edit wars. Here we try to recognize some general features based on numerical properties of 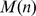 and its derivative, and categorize the articles accordingly. We applied a maximum detection tool to the smoothed derivative curve to locate both the hot periods of wars and the ‘consensus reached’ situations where the derivative of 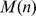 is very small or zero. Based on the statistics of the war and consensus periods, we categorize articles into three main categories.

##### *a) Consensus*

The common scenario for the cases where at the end consensus is reached is the following. Usually growth starts slowly and with an increasing acceleration until it reaches a maximum speed of growth. Afterwards, when the hot period of war is passed, the growth rate decreases and consensus is reached, where 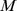 does not, or only very slightly, increases upon the next edits. We do not offer a mathematical model for such growth here, but we note that a Gompertz function 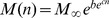 (with 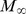 being the final value of 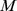, and 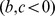 are the displacement parameter and growth rate respectively) offers a reasonable fit (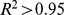) for almost all 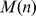 in this category (see Figure 13 for an example). In general, the Gompertz function fares better than sigmoid because it does not force symmetry around the initial and the final asymptote, and the literature such as [59] suggests it is a more appropriate model for growth in a confined space. We leave the matter of how controversiality becomes a consumable resource for future research, but we find it quite plausible that certain articles can become so well polished that it becomes extremely hard to pick a fight about them.

##### *b) Sequence of temporary consensuses*

The common feature of the articles in this category is sequential appearance of war and peace periods in a quasi-periodic manner. After the first cycle of war and consensus as described in (a), internal or external causes initiate another cycle. Exogenous changes happen completely randomly, but the endogenous causes may be contributed by a simple mechanism such as a constant influx of new editors, who are not satisfied with the previously settled state of the article (see Figure 14 for an example).

We do not have the means to make the required systematic distinction between internal end external causes (manual evaluation is too expensive, auto-detection would require too much world knowledge). Therefore, we created a limited sample of 44 articles, which are entirely about solid concepts and facts, in order to measure the periodicity of endogenous controversies. Figure 15 gives a histogram of the distance (number of edits) between two successive war periods. We obtain a mean value of 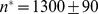.

##### *c) Never-ending wars*

In the evolution of the articles in this category no permanent, or even temporary, consensus gets ever built. Articles describing intrinsically highly controversial/hot topics tend to belong in this category (see Figure 16 for an example).

We sorted all articles with 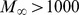 in one of the categories (a–c) and calculated the relative share of each category at a given 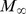. The results are shown in Figure 17. Keeping in mind that less than 1% of WP pages is controversial (some 12 k out of 3.2 M in the original data set have 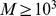), we see that only a small fraction of these fit the ‘multiple consensuses’ category (b), with the majority fitting rather clearly in the two polarly opposed classes (a) and (c). Quite as expected, with the growth of 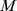 category (a) dies out, since consensus is reached, and only articles in the never-ending war category remain. While in earlier research we set the controversiality threshold at 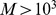, Figure 17 is suggestive that there is hope for consensus by natural process as long as 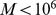, while the remaining subjects are truly ‘bad apples’, and it is a credit to the WP community that such cases are kept to a minuscule proportion of less than 100 in the entire set of 3.2 M articles.

### Talk pages and Conflict Resolution

Talk pages (also known as a discussion pages) in WP are supposed to be pages where editors can discuss improvements to an article or other Wikipedia page [60]. Each article could have its own talk page in addition to user talk pages, which host more personal discussions. In Ref. [34], case studies of talk pages of 58 selected articles were reported – the authors concluded that a considerable portion of talk pages are dedicated to discussions about removed materials and controversial edits. In the following, we report our preliminary results on how well talk pages reflect editorial wars and to what extent they help in resolving disputes.

#### Talk page length

Those familiar only with the English WP may come to the conclusion that the length of talk pages associated to each article could provide a simple, direct measure of controversiality, especially as the whole mechanism of talk pages was invented to channel controversies. As can be seen from Figure 18, article length and talk page length are distributed quite differently, with the log-normal providing a very good fit for article length (and a reasonable genesis as a multiplicative process with a left barrier, here the minimum length of an article, [61]) but not for talk page length, which is no surprise, since there is no left barrier for the lengths of the talk pages. (We mention that the total number of edits on an article has also been argued to be log-normally distributed [33].).

As can be seen from Figure 19, the correlation between article and talk page length is not very strong (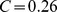) – the most natural hypothesis is that the discrepancy is caused by the fact that articles of the same length can nevertheless have different degrees of controversiality. In the English WP talk page length correlates reasonably well (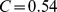) with 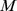 (see Figure 20), yet in other WPs, talk pages are used far less: for example, in the Hungarian WP editors solve their conflicts directly on the pages, changing and reverting the versions which they do not like, generally without any talk or arguments, while in the Spanish WP (which has the longest talk pages after normalization by article length) or the Czech WP, the talk pages are generally more cooperative. According to this result, it becomes evident that the philosophy of “talk before type” [62] is not truly followed in practice. Depending on culture, talk pages can be reflective of the conflicts and edit wars, but they do not act as a dampening mechanism.

#### Discussion networks

We begin by some qualitative observations that emerge from the manual study of the networks of editorial interactions such as depicted in Figure 21. It appears that the discussions on talk pages are dominated by continual back and forth between those editors who hardly change their opinion. In contrast to other social networks, clusters beyond pairs are rare. Editors joining the discussion at later stages have very little chance of becoming one of these high-activity editors. Less active editors tend to address the more active ones rather than each other – from studying the text one gets the distinct impression that they do not consider the other low activity editors worthy of commenting upon. Also, the less prolific editors appear more negative, more fierce, hysterical in tone, sometimes downright irrational. Debates rarely conclude on the basis of merit: typically they are ended by outside intervention, sheer exhaustion, or the evident numerical dominance of one group.

Based on these observations we hypothesized that most of the editorial war is carried on by only a few editors. To check this, we have looked at the *top 5* *ratio*, 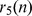 defined as 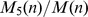 where 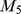 is the value of 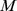 only considering the contributions of the top 5 pair of editors (ranked by their mutual reverts) among all the editor pairs of the article. In Figure 22, values of 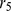 calculated from the whole sample are visualized as a function of 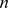 and 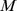. The color code is corresponding to the average value of 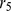 for the points located in each cell. Perhaps surprisingly, this number is quite large (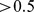)) for many articles and for long periods of the article’s life, meaning that a large fraction of the whole war is indeed caused by a small number of fighting pairs. 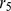 becomes smaller than 0.5 only for the articles which are already in the controversial region (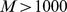) and were edited many times (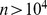). Smaller values of 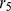 can be observed only in the articles which belong to the category of never-ending wars. In these articles, many different editors have fought at different periods of time, and a steady flow of replacement armies keeps the article always far from equilibrium.

In conclusion, we showed that conflicts and editorial wars, although restricted to a limited number of articles which can be efficiently located, consume considerable amounts of editorial resources. Moreover, we observed that conflicts have their own temporal fingerprint which is rooted in memory effects and the correlation between edits by different editors. Finally, we demonstrated that, even in the controversial articles, often a consensus can be achieved in a reasonable time, and that those articles which do not achieve consensus are driven by an influx of newly arriving editors and external events. We believe that these empirical results could serve as the basis of more theoretical agent-centered models which could extend beyond the WP development process to other large-scale collective and collaborative problem-solving projects.

## Supporting Information

Figure S1
**Burst statistics for users’ editorial activity.** Upper panel: distribution of time interval between two successive edits made by a certain user on any article 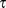. Middle panel: 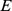, the number of events in the bursty periods separated by a silence window of 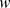. Lower panel: autocorrelation function 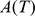 for the editing time train of individual users.(TIFF)

Text S1 **Details of classification experiments.** More details of peaceful/controversial classification experiments based on human judgment are given.
(PDF)
