# Supplementary figures and images for: Dynamics of Conflicts in Wikipedia
Source: PLoS One. 2012 Jun 20;7(6):e38869. doi: 10.1371/journal.pone.0038869 (PMC3380063; doi:10.1371/journal.pone.0038869)

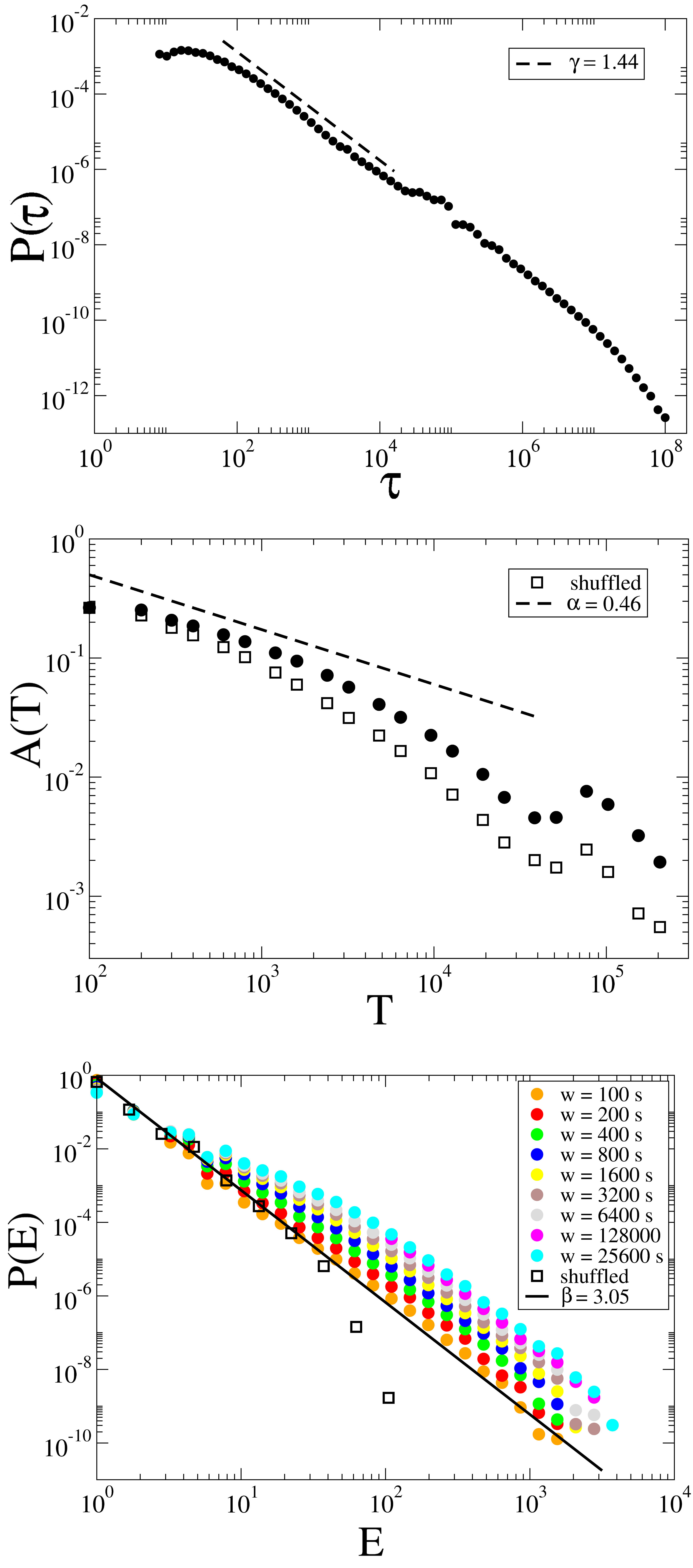

Supplement: Figure S1 — Burst statistics for users’ editorial activity. Upper panel: distribution of time interval between two successive edits made by a certain user on any article . Middle panel: , the number of events in the bursty periods separated by a silence window of . Lower panel: autocorrelation function for the editing time train of individual users. (TIFF) [file pone.0038869.s001.tiff]
