# Supplementary material for: Dynamics of Conflicts in Wikipedia
Source: PLoS One. 2012 Jun 20;7(6):e38869. doi: 10.1371/journal.pone.0038869 (PMC3380063; doi:10.1371/journal.pone.0038869)
Supplement: Text S1 — Details of classification experiments. More details of peaceful/controversial classification experiments based on human judgment are given. (PDF) [file pone.0038869.s002.pdf]

Text S1: Details of classification experiments

During our work we frequently solicited human judgment on how peaceful or controversial certain pages appear to the observer. Rather than relying on the everyday meaning of *peaceful* versus *controversial*, we have instructed the judges to use several confluent criteria that we list here in no particular order.

- **Rant:** truly hysterical behaviour without much content, usually against someone or a group of editors
- **Help:** asking for outside help or the help of other editors
- **Vote:** voting, merging, moving or talking about these
- **Prot:** talking about protecting the page
- **Ban:** talking about banning somebody
- **Warn:** warning about some bad consequences if somebody does something
- **Command:** ordering, rather than asking, somebody to do, and especially to not do, something
- **Rev:** talking about reverts
- **Irony:** ironizing over the others. This could be very rude when it is observed jointly with other symptoms like accusation but could be quite sophisticated used by senior editors who are generally very neutral (not accusing, warning etc.). Same tag used for any form of malicious joking at the expense of others
- **Acc:** accusing somebody in the talk of POV, not reading comments, not understanding them, repeating the same arguments etc.
- **Rep:** talk about repeating the same problems or arguments over and over again
- **Comp:** complaining about anything, generally about the others' behavior
- **Emo:** using emotion related words in argumentation: e.g. *I strongly disagree, are you kidding?* etc.
- **Formal:** using formal naming style: e.g. referring to other user as *User Tabib* or *Mr. Tabib* rather than the usual *Tabib*

- **UTCite:** citing user talk pages
- **SelfSupp:** writing something than adding some new comments immediately
- **Stepwise:** answering former comment line by line

Needless to say, judging many of these criteria is also a highly subjective matter: who is to say whether a certain passage is ironic, whether it truly constitutes a warning, or whether it is a rant? Nevertheless, human judges showed quite significant correlation with one another (and with the machine-generated M score, as seen e.g. in Fig 2). In the body of the paper we reported on experiments that took the high-conflict sample from the range $10,000 < M < 70,000$ and the low-conflict control from the range $100 < M < 150$, i.e. on the average a factor of 280 between the two groups.

To test how well humans do, we constructed a less sharply separated sample of 30 pages with $M \approx 50$ for low conflict and 30 pages with $M \approx 2,500$ for high conflict, i.e. on the average a factor of 50 between the two groups. We had four human judges, instructed in the above criteria, who had to check all 60 pages given to them in random order. The most peace-leaning judge found 33 instances of controversality, the most war-leaning judge found 39 (in accordance with our design of the measure M, which aimed at generating fewer false positives than false negatives). Remarkably, the correlation between the most lenient and the most strict judge is still $r = 0.92$, with a κ coefficient of 0.79, at the high end of what is generally considered 'substantial' agreement. (This is the worst case: the correlation between the most war-leaning judge and the other two judges is 0.935 and 0.987, Cohen's κ is 0.82 and 0.96, usually considered 'almost perfect' agreement.)

Not only are the opinions of the judges correlated, they show evident graduality: if the most peace-leaning judge declares a page controversial, the other three will declare it controversial with probability $1, 0.9$, and 1 respectively, and if the most war-leaning judge declares it peaceful the others will also do so with probability $1, 1$, and 0.9. The result is a manifestly bimodal distribution, where if we assign one point for each vote of controversality, 49 pages receive 0 or 4 points, another 10 receive 1 or 3 points, and only 1 page in the entire sample of 60 receives 2 points, truly splitting the judges. Were the judgments uncorrelated, we would expect to see the exact opposite picture, with most pages (22.5 out of 60) receiving a score of 2, 15-15 receiving 1 and 3, and less than 8 receiving some extreme score.

Based on this level of interobserver agreement there cannot be any doubt that manual classification of WP pages as peaceful vs. controversial can be done quite reliably. This is not to say that the

process is completely repeatable, but even a lot simpler classification tasks, such as deciding weather a character is an *l* or a *1*, or whether a word in some context is a noun or a verb, tend to fall shy of r or $\kappa > 0.95$. However, we relied on human judgment only to the extent it was necessary to create, calibrate, and validate our controversy measure M, all subsequent results use M directly and are therefore fully replicable.

It is perhaps worth pointing out that our primary interest is not with the human concept of controversality, but rather with the wars themselves. Accordingly, we have not made an all out effort to minimize the misclassification rate of M, and there is no doubt that by including more factors (ranging from talk page length to the number of times banning somebody is discussed) a much more sensitive measure could be developed. However, as M correlates nearly as well with human judgment as the least correlated humans correlate with one another, $r = 0.80$ vs. $r = 0.85$, there is no reason to believe that a more sensitive measure would substantially alter the picture presented here.
